# Antibacterial Activity of a Trace-Cu-Modified Mg Alloy in Simulated Intestinal Fluid

**DOI:** 10.3390/jfb16090344

**Published:** 2025-09-12

**Authors:** Baiyun Zhong, Zemeng Wei, Yi Yao, Lixun Jiang, Manli Zhou, Jinping Li, Weidong Liu, Xin Li, Ming-Chun Zhao

**Affiliations:** 1 Department of Clinical Laboratory, Xiangya Hospital, Central South University, Changsha 410008, China; zby@csu.edu.cn (B.Z.); xycsuhn@163.com (M.Z.); 2 National Clinical Research Center for Geriatric Disorders, Xiangya Hospital, Central South University, Changsha 410008, China; weidong.liu@csu.edu.cn; 3 Department of General Surgery, Xiangya Hospital, Central South University, Changsha 410008, China; wzmeng163@163.com (Z.W.); hy_zirui@163.com (L.J.); 4 Department of Gastroenterology, First Medical Center, PLA General Hospital, Beijing 100036, China; yaoyi303@vip.126.com (Y.Y.); lijinping316@163.com (J.L.); 5 School of Materials Science and Engineering, Central South University, Changsha 410083, China; 243101040@csu.edu.cn

**Keywords:** trace-Cu-modified Mg alloy, intestinal environments, biodegradation, antibacterial activity, cytocompatibility

## Abstract

Mg alloys hold promise for biodegradable gastrointestinal implants, but most evaluations rely on simplified media like Hank’s solution, which lacks organic components and fails to replicate the acidic-to-alkaline transition of intestinal fluid, risking underestimation of biodegradation rates and clinical relevance. This work investigated a trace-Cu-modified Mg alloy (Mg-0.05Cu) in simulated intestinal fluid (SIF) versus Hank’s solution. Microstructural analysis confirmed Mg_2_Cu intermetallic phases as Cu reservoirs. Electrochemical and immersion tests revealed significantly accelerated biodegradation in SIF, due to its disruption of protective layer formation, sustaining loose biodegradation products. The biodegradation rate of the trace-Cu-modified Mg alloy in SIF was consistent with reported values for Mg alloys in similar media, as was that in Hank’s solution. Remarkably, Mg-0.05Cu exhibited potent antibacterial activity against *E. coli*, achieving 99.3% eradication within 12 h and 100% elimination by 24–48 h, alongside excellent cytocompatibility with L929 cells (>95% viability). This efficacy arose from the synergistic Cu^2+^ release and high-pH microenvironment. These findings demonstrate that trace Cu alloying in high-purity Mg balances rapid antibacterial action with controlled biodegradation in a physiologically relevant SIF. This positions Mg-0.05Cu as a highly promising candidate for practical applications, such as biodegradable intestinal stents, anti-adhesion barriers, anastomosis rings, and anti-obesity devices, where rapid infection control and predictable degradation are critical for clinical success. This work underscores the importance of using biomimetic media for evaluating gastrointestinal implants and establishes Mg-0.05Cu as a promising strategy for developing infection-resistant biodegradable devices.

## 1. Introduction

Digestive system disorders represent a significant disease burden, with gastrointestinal surgery serving as a common therapeutic intervention. Consequently, implantable medical devices for gastrointestinal applications, such as biliary/intestinal stents, hemostatic clips, and anastomotic staples/rings, have gained substantial interest [1,2,3,4]. Currently, 316L stainless steel and Ti alloys dominate this field [5,6,7,8]. However, these materials present notable limitations. 316L stainless steel, despite its slow corrosion rate, remains susceptible to corrosion primarily induced by bacteria and high chloride concentrations. Corrosion releases metal ions that may cause allergic reactions, cytotoxicity, carcinogenicity, pro-inflammatory effects from corrosion byproducts (e.g., nickel, chromium, and molybdenum ions), gastrointestinal tissue damage from peristaltic traction forces, and significant MRI imaging artifacts. Ti alloys like TiNi are widely used for their superelasticity and shape memory properties but contain nickel, which poses high risks of metal allergy and carcinogenicity. Critically, both 316L stainless steel and Ti alloys are nondegradable, necessitating secondary removal surgeries [9]. Mg and its alloys have emerged as promising alternatives to permanent implants due to their excellent biocompatibility and biodegradability [10,11,12]. Their degradation products are generally nontoxic and can be metabolized or excreted by the body, thus avoiding long-term foreign body reactions. Moreover, magnesium ions released during degradation have been shown to promote osteogenesis and wound healing, further enhancing their suitability for biomedical applications [10]. However, gastrointestinal environments exhibit greater complexity than typical body fluids, including dynamic pH variations, enzymatic activity, and mechanical peristalsis [13]. Although numerous studies have evaluated Mg alloy biodegradation in site-specific physiological media such as simulated body fluid (SBF) or Hank’s solution, research on Mg alloy biodegradation in gastric and intestinal fluids remains limited. For instance, studies indicate that the degradation rate of the Mg-2Zn alloy in intestinal fluid can exceed 10 mm/year, significantly higher than in conventional media, underscoring the need for more physiologically relevant testing environments [13].

Furthermore, existing gastrointestinal implants often lack reliable anti-inflammatory and antibacterial properties, leading to high infection rates. Surgical site infections, where bacterial colonization causes implant failure, tissue inflammation, and systemic complication, pose major clinical concerns. While Mg alloys inherently generate alkaline microenvironments (via biodegradation) that modestly inhibit bacteria, physiological buffering rapidly neutralizes this effect, diminishing antibacterial efficacy [14,15]. To address this limitation, strategic alloying with antibacterial elements has been explored. Copper (Cu), in particular, is known for its broad-spectrum antibacterial properties and serves as an ideal modifier for Mg, enabling the development of functional antibacterial Mg-Cu alloys [16,17,18]. Previous research has demonstrated that Mg-Cu alloys with Cu content above 0.5 wt.% can achieve >90% antibacterial efficiency against common pathogens such as Escherichia coli (*E. coli*) and Staphylococcus aureus (*S. aureus*) [17]. However, high Cu content also accelerates degradation via microgalvanic corrosion, leading to premature loss of mechanical integrity and potential cytotoxicity [16].

Nevertheless, the biodegradation behavior and antibacterial performance of Mg-Cu alloys in harsh dynamic gastrointestinal environments remain inadequately investigated. Most evaluations rely on simplified media like Hank’s solution, which lacks organic components and fails to replicate the acidic-to-alkaline transition of intestinal fluid, risking underestimation of biodegradation rates and clinical relevance. Additionally, Cu incorporation typically accelerates Mg alloy biodegradation by promoting microgalvanic corrosion via Cu-containing intermetallics [16,17], particularly in harsh gastrointestinal conditions. Rapid biodegradation may exacerbate cytotoxicity and hinder clinical translation. Therefore, this study incorporates only trace Cu (0.05 wt.%) into high-purity Mg, a dual strategy designed to leverage high-purity Mg’s intrinsically superior biodegradation resistance while minimizing biodegradation acceleration and avoiding other toxic alloying elements. This approach aims to achieve a balance between antibacterial functionality and controlled degradation.

This work systematically characterizes the microstructure, biodegradation kinetics, biodegradation mechanisms, and antibacterial functionality of trace-Cu-modified Mg alloys, focusing on two pivotal aspects: (1) divergent biodegradation behavior in simulated intestinal fluid (SIF) versus Hank’s solution; (2) antibacterial efficacy against Escherichia coli (*E. coli*), a prevalent opportunistic pathogen. By elucidating biodegradation–antibacterial synergies in physiologically relevant intestinal environments, this study advances the design of Mg-based implants with inherent infection-controlling capabilities. It underscores the necessity of biomimetic media for gastrointestinal device evaluation and establishes trace Cu alloying as a viable strategy to enhance the safety and functional longevity of biodegradable Mg in clinical applications.

## 2. Experiments

### 2.1. Material Preparation

The trace-Cu-modified Mg alloy ingots were produced in an electronic resistance furnace by melting high-purity Mg (>99.99 wt.%) and Cu (>99.99 wt.%), and then cast into a circular water-cooled copper mold with CO_2_/SF_6_ mixture gas as a protection atmosphere. Table 1 presents the measured chemical compositions of the as-cast alloy, designated as Mg-0.05Cu. Samples were sectioned from an as-cast ingot and subjected to solid solution treatment at 510 °C for 10 h with the sample surface protected by carbon powders, followed by water quenching. More details are described elsewhere [19,20]. For simplicity, the as-cast condition and solid solution treatment condition are subsequently abbreviated as the AC condition and the SS condition, respectively.

### 2.2. Microstructural Characterization

Specimens from the AC and SS conditions were prepared using conventional metallographic methods: they were successively ground with 1200-grit SiC paper, polished with 0.5 µm diamond paste, rinsed in distilled water, dried under warm airflow, and etched in 4% nital.

Microstructures were characterized using optical microscopy (micro) and scanning electron microscopy (SEM; ZEISS EVO 18, Berlin, Germany). Phase distribution was analyzed by energy-dispersive X-ray spectroscopy (EDS) equipped on the SEM. Constituent phases were identified via X-ray diffractometry (XRD; D/max 2550, Tokyo, Japan) using Cu Kα radiation (40 kV, 30 mA) with a scan rate of 8°/min.

### 2.3. Electrochemical and Immersion Tests

Electrochemical and immersion tests were conducted at 37 °C in SIF containing 1% trypsin, with comparative testing in Hank’s solution.

Electrochemical tests were performed using an electrochemical workstation (Zahner IM6ex, Kronach, Germany) with a standard three-electrode system. A saturated calomel electrode (SCE), platinum electrode, and sample served as reference, counter, and working electrodes, respectively. Samples for potentiodynamic polarization were epoxy-encapsulated to expose a 10 mm × 10 mm surface area to 500 mL of solution. Surfaces were prepared by successive mechanical grinding with 1200-grit SiC paper followed by distilled water rinsing. All potentials were measured with reference to the SCE. After open-circuit potential stabilization, polarization curves were recorded by scanning from a cathodic potential to an anodic potential at a scan rate of 0.5 mV/s. Electrochemical impedance spectroscopy (EIS) measurements used a 10 mV amplitude with the frequency ranging from 100 kHz to 0.01 Hz.

Immersion tests followed ISO 10993 at room temperature with a surface-area-to-solution-volume ratio of 1.25 cm^2^/mL. Specimens were prepared identically to electrochemical samples. Initial weights were recorded pre-immersion. Post-immersion specimens were ultrasonically cleaned in acetone and distilled water to remove biodegradation products, then dried, and weighed. Surface morphologies and biodegradation products were characterized by SEM-EDS.

### 2.4. Extract Preparation

Samples were sectioned into uniform dimensions (Ø10 mm × 2 mm). Surfaces were successively mechanically ground with 1200-grit SiC paper and polished. Samples were ultrasonically cleaned in ethanol and deionized water (15 min each), and then sterilely dried. Following ISO 10993 [9,10,11,12,13], extracts were prepared at a surface-area-to-volume ratio of 1.25 cm^2^/mL. Sterilized samples were immersed in sterile phosphate-buffered saline (PBS) for antibacterial testing or Alpha Minimum Essential Medium (α-MEM) supplemented with 10% fetal bovine serum (FBS) and 1% penicillin–streptomycin for cytocompatibility assays. All extractions were performed at 37 °C for 24 h with gentle agitation. Post-incubation, extracts were filtered through 0.22 μm sterile membranes and stored at 4 °C.

### 2.5. Antibacterial Evaluation

The antibacterial activity of specimen extracts was evaluated using *E. coli* (ATCC 25922) as a model strain. Fresh bacterial suspensions were prepared in sterile PBS and adjusted to ~1 × 10^6^ colony-forming unit (CFU)/mL. Bacterial suspension (100 μL) was mixed with 900 μL of specimen PBS extract for testing. A blank control consisted of bacterial suspension mixed with pure PBS. All specimens underwent static incubation at 37 °C for 24 h. Post-incubation, suspensions were serially diluted with PBS, plated on nutrient agar, and incubated at 37 °C for 24 h. The 10^5^ dilution (0.1 mL plated) was used for CFU/mL calculation and antibacterial rate analysis. Photographs of the plates were taken to observe the growth of bacterial colonies. Each experiment was conducted in triplicate to ensure reproducibility.

### 2.6. Cytocompatibility Assay

L929 fibroblasts were seeded in 24-well plates at 2 × 10^4^ cells/well and cultured in α-MEM supplemented with 10% FBS and 1% penicillin–streptomycin. After 24 h incubation, media were replaced with either specimen extract or fresh medium (blank control). Following 1- and 3-day incubations, cell viability was assessed using live/dead staining: Cells were PBS-washed, and then incubated with calcein-AM/propidium iodide (PI) working solution (37 °C, 30 min). Fluorescent images were acquired using an inverted fluorescence microscope (Leica DMI8, Heidelberg, Germany). Cell proliferation was quantified via the Cell Counting Kit-8 (CCK-8) assay. At each timepoint, 10 μL CCK-8 reagent was added to 100 μL culture medium per well. After 2 h incubation (37 °C), absorbance was measured at 450 nm using a microplate reader (Multiskan FC, Thermo, Waltham, MA, USA). Each experiment was performed in triplicate under aseptic conditions, with results expressed as the mean ± standard deviation.

## 3. Results

### 3.1. Microstructures

**Figure 1:**
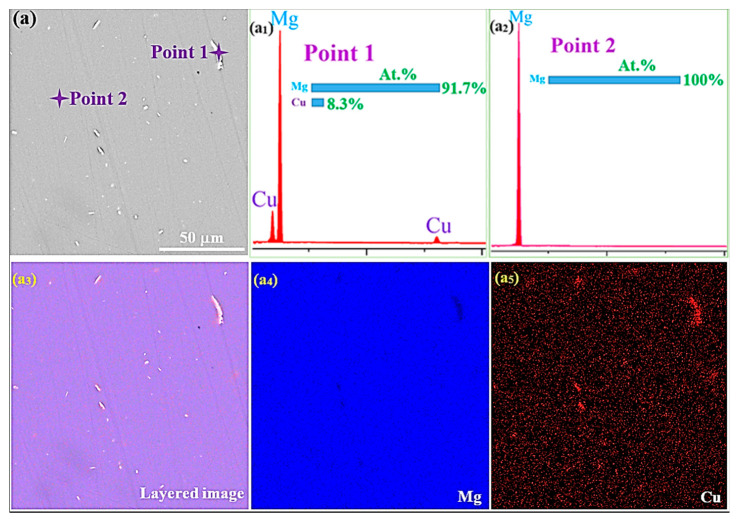

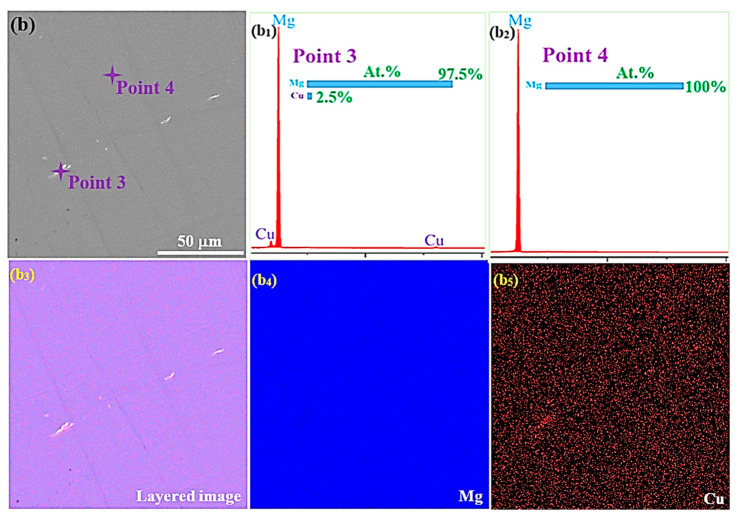
SEM-EDS data for AC condition: (**a**) SEM image, (**a_1_**,**a_2_**) EDS spectra for points 1 and 2 in (**a**), and (**a_3_**–**a_5_**) EDS elemental maps of regions in (**a**); and SS condition: (**b**) SEM image, (**b_1_**,**b_2_**) EDS spectra for points 3 and 4 in (**b**), and (**b_3_**–**b_5_**) EDS elemental maps of regions in (**b**). Note: points 1 and 3: second phase; points 2 and 4: matrix.

Figure 1 shows SEM-EDS data of the AC and SS conditions, revealing compositional distributions in the second-phase particles and matrix grains. The SEM image of the AC condition showed a gray primary α-Mg matrix and bright second-phase particles that were granular or short-rod-like in shape, primarily distributed within the matrix (Figure 1a). In contrast, the SEM image of the SS condition displayed the gray primary α-Mg matrix and significantly fewer bright second-phase particles (Figure 1b), indicating a marked reduction in secondary-phase particles after solution treatment due to the decomposition of these phases into the matrix. EDS spectra for points 1 and 2 marked in Figure 1a (Figure 1(a_1_): point 1, second phase; Figure 1(a_2_): point 2, matrix) and for points 3 and 4 marked in Figure 1b (Figure 1(b_1_): point 3, second phase; Figure 1(b_2_): point 4, matrix) indicated that the second-phase particles in both the AC and SS conditions were Cu-rich. These particles exhibited significantly higher Cu content than the corresponding matrix. EDS elemental maps of the regions shown in Figure 1a,b revealed that Mg was dispersed throughout the matrix, while Cu was primarily distributed within the second phases (Figure 1(a_3_): layered image; Figure 1(a_4_): Mg distribution; Figure 1(a_5_): Cu distribution for the AC condition; Figure 1(b)_3_: layered image; Figure 1(b_4_): Mg distribution; Figure 1(b_5_): Cu distribution for the SS condition). EDS analysis confirmed that the secondary phase in both conditions was composed of Mg and Cu. According to the Mg-Cu binary phase diagram, Mg-Cu alloys undergo a eutectic reaction at 485 °C, decomposing into α-Mg and Mg_2_Cu intermetallics during cooling [21]. Therefore, it is reasonable to conclude that the secondary phase is Mg_2_Cu intermetallics. Notably, the high Mg concentration in the secondary phase, reflected by the high atomic Mg/Cu ratios derived from EDS analysis (11:1 in Figure 1a_1_; 39:1 in Figure 1(b_1_), is likely attributed to the presence of α-Mg within the eutectic structure (Mg_2_Cu + α-Mg).

**Figure 2:**
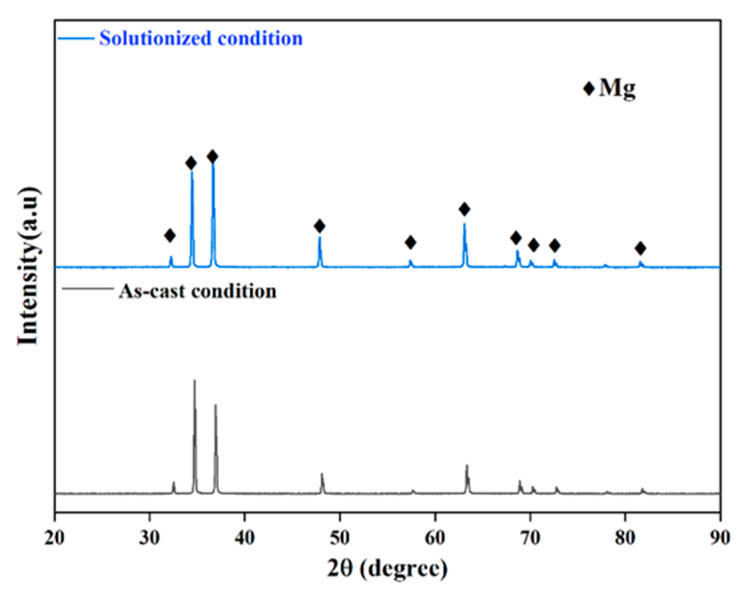
XRD patterns of the AC and SS conditions.

Figure 2 shows the XRD patterns of the AC and SS conditions. Notably, the Mg_2_Cu peaks cannot be identified in the XRD pattern due to the low concentration of Cu (0.05 wt.%) in the experimental alloy and the limitation of the instrument. Only the peaks of Mg can be observed in the XRD pattern.

Given the above, the microstructure under the SS condition is more uniform than that of the AC condition, as evidenced by the significantly reduced second-phase distribution. Consequently, subsequent biological experiments, including antibacterial and biocompatibility tests, were conducted using the SS samples.

### 3.2. Degradation

**Figure 3:**
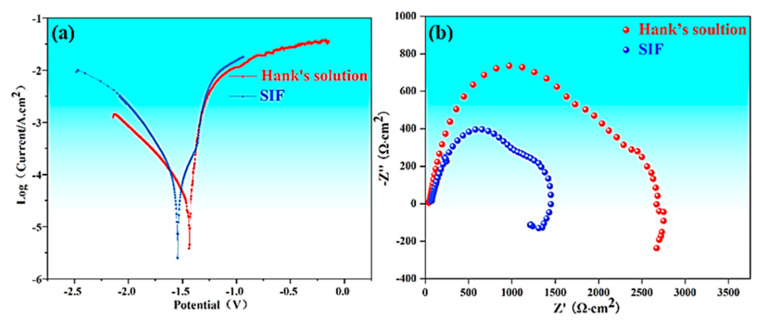
Electrochemical data: (**a**) polarization curves; (**b**) Nyquist plots.

Figure 3a compares the potentiodynamic polarization curves of the SS condition in SIF and Hank’s solution. Both curves are similar. The cathodic branch corresponds to hydrogen reduction reactions, while the anodic branch reflects Mg matrix dissolution. The corrosion potential (*E*_corr_) tested in SIF was somewhat more negative than that tested in Hank’s solution. A more negative *E*_corr_ in SIF indicates a greater tendency for the Mg matrix to corrode in this specific environment. Essentially, this is because the increase in the negative value of the electrode potential makes the Mg matrix more prone to losing electrons and undergoing oxidation reactions. The corrosion current density (*i_corr_*) was evaluated from the polarization curves by Tafel extrapolation using the linear cathodic branch. The *i_corr_* value in SIF was 239 μA/cm^2^, while that in Hank’s solution was 61 μA/cm^2^; the former was almost four times higher than the latter. This indicates that the dissolution reaction of the Mg matrix in SIF occurs at a much higher rate than that in Hank’s solution. For easier comparison, the biodegradation rates (*P_i_*) for both solutions are calculated from electrochemical data using Equation (1) [22]:

*P_i_* (mm/year) = 22.85 *i_corr_* (mA cm^2^) (1)

The *P_i_* in SIF was ~5.5 mm/year, while that in Hank’s solution was ~1.4 mm/year. The *P_i_* obtained in SIF was demonstrated to be higher than that in Hank’s solution.

Figure 3b compares the EIS Nyquist plots of the SS condition in SIF and Hank’s solution. The EIS measurements are usually used to assess the protective properties of the naturally formed corrosion layer, which is directly relevant to the material’s degradation performance. In the case of the present Mg-0.05Cu alloy, the corrosion layer that forms in physiological environments somewhat functions as a protective coating. Therefore, using EIS to evaluate the stability and degradation of this layer provides valuable insight into the material’s overall degradation behavior. The Nyquist plots presented similar shapes in SIF and Hank’s solution, consisting of a single capacitive loop with varying diameters in the measured frequency range. The diameter of the capacitive loop correlates with polarization resistance: a smaller diameter indicates lower polarization resistance and a higher degradation rate [23,24,25]. The smaller capacitive arc diameter corresponding to SIF signifies a faster electrochemical process, aligning with the above-mentioned higher *P_i_* obtained in SIF than in Hank’s solution.

**Figure 4:**
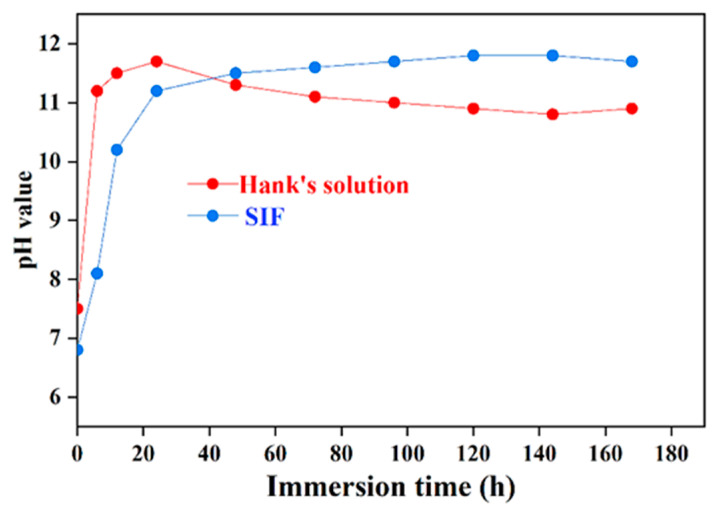
The pH variation curves of SIF and Hank’s solution during immersion for 168 h.

Figure 4 shows the pH variation curves of SIF and Hank’s solution during immersion for 168 h (7 days). It can be observed that the pH change trend is just slightly different in the different solutions. In Hank’s solution, the pH value surged sharply from the initial 7.5 to ~10.5 after 3 h of immersion, reaching its peak of 11.7 after 24 h. Subsequently, with prolonged immersion time, the pH value gradually decreased and eventually stabilized to ~11. The initial pH increase is primarily due to the direct contact between the specimen surface and Hank’s solution at the beginning of the immersion test. Anions such as Cl^−^ and CO_3_^2−^ in Hank’s solution react with the Mg matrix, causing rapid biodegradation of the specimen surface. This process generates Mg(OH)_2_ precipitates on the surface and leads to the accumulation of OH^−^ ions. The anodic reaction and the cathodic reaction are given by Equation (2) and Equation (3), respectively, and the overall biodegradation reaction is given by Equation (4). The higher pH value indicated a higher biodegradation rate.

(2) $$Mg-2e↔{Mg}^{2+}.$$

(3) $$2H_{2}O+2e↔H_{2}↑+2{OH}^{-}$$

(4) $$Mg+2H_{2}O↔Mg{\left(OH\right)}_{2}+H_{2}$$

As immersion time increases, the biodegradation reaction continues, explaining the continuous pH rise up to 24 h. During the middle and later stages of immersion, the pH begins to decline. Although biodegradation persists, white products like hydroxyapatite form on the specimen surface. These biodegradation products isolate the specimen surface from direct contact with Hank’s solution. This reduces the biodegradation rate, slows down OH^−^ accumulation, and causes the pH to decrease and relatively stabilize. In the SIF, the initial pH is 6.8, creating an acidic environment that accelerates biodegradation. Consequently, the pH shifts from acidic to neutral during the early immersion stage. Since the biodegradation rate in SIF is significantly higher than in Hank’s solution, a sharp pH surge occurs within a short time as immersion progresses, eventually stabilizing to 11.8. Comparing the stabilized pH values in both solutions, the stabilized value in SIF is higher than in Hank’s solution. This can be attributed to a higher accumulation of OH^−^ ions (indicating a higher biodegradation rate) in the former after stabilization. This experimental result is consistent with the findings obtained from electrochemical tests.

**Figure 5:**
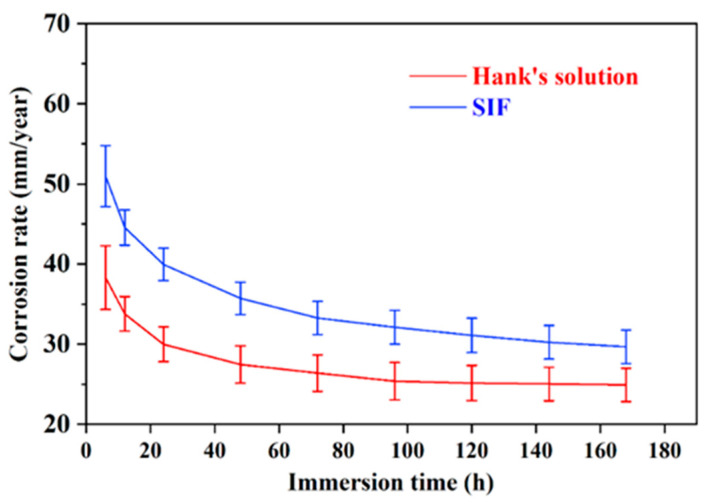
The variation curves of the biodegradation rate (*P*_w_) in SIF and Hank’s solution.

The mass loss measure through immersion tests was employed to monitor the periodical biodegradation rate for 168 h while replacing the immersion fluid every 6 h. Figure 5 presents the variation curves of the biodegradation rate (*P*_w_) in SIF and Hank’s solution. The *P*_w_ was calculated based on weight loss data using Equation (5) [22]:

*P*_w_ (mm/year) = 2.10 Δ*W* (weight loss rate) (mg cm^−2^/day) (5)

The initial *P*_w_ is high, and at a later stage, the *P*_w_ decreases; i.e. the biodegradation rate decreases along with immersion time. Notably, the *P*_w_ in SIF is always higher than that in Hank’s solution for every specific immersion time during the whole immersion process.

The biodegradation rates determined from the independent measurement of polarization curves (*P_i_*) and immersion tests (*P*_w_) are different. The *P_i_* is apparently lower, as demonstrated above and frequently observed in studies [17,26]. However, both methods exhibit the same trends: the *P_i_* in SIF is higher than that in Hank’s solution, as is the *P*_w_. The *P_i_* relates to the biodegradation onset, while the *P*_w_ reflects the average biodegradation rate over a considerable period following onset. During immersion testing, the biodegradation rate was influenced by the surface biodegradation products. The biodegradation rate from immersion testing is more practical. Notably, the *P_i_* and *P*_w_ values of the trace-Cu-modified Mg alloy in SIF are consistent with reported values for Mg alloys in similar media, as were those in Hank’s solution [3,20,27,28,29,30]. This agreement provides confidence in the present experimental measurements.

**Figure 6:**
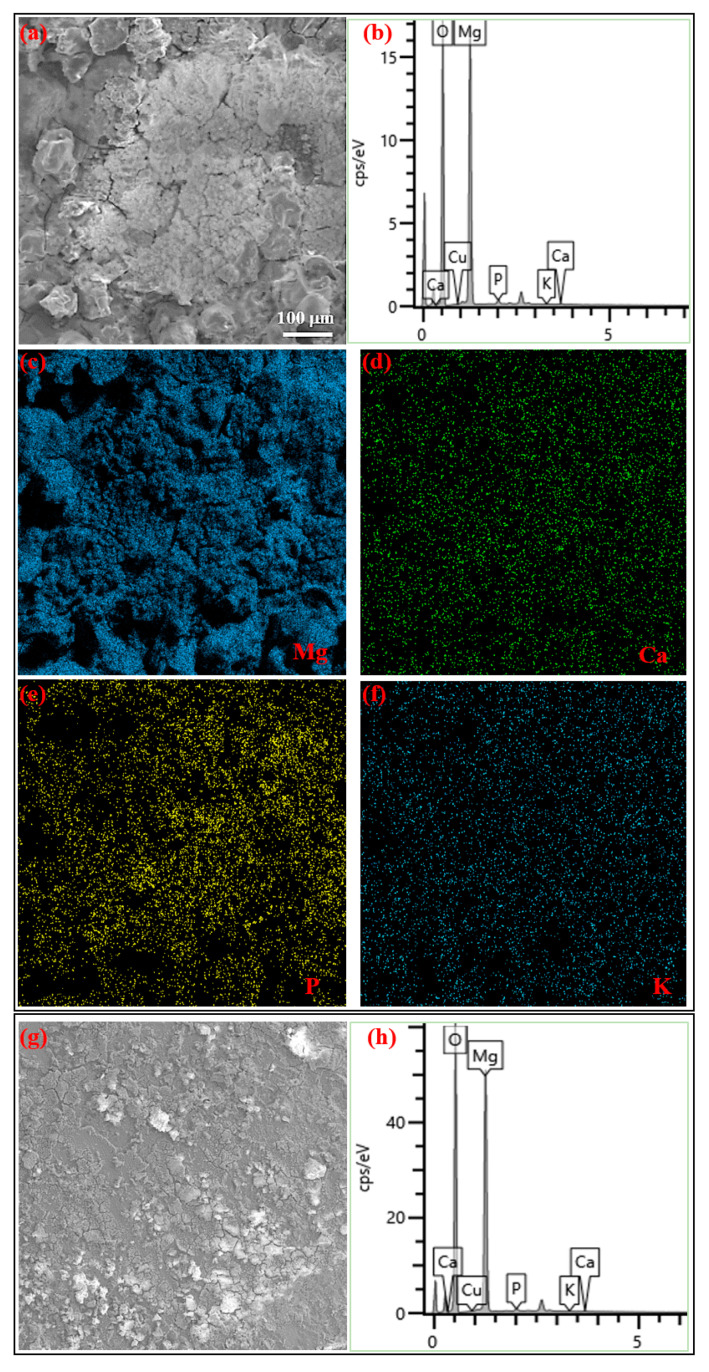
Biodegradation morphology and products after 7-day immersion in SIF (**a**–**f**) and Hank’s solution (**g**,**h**).

Figure 6 shows the biodegradation morphology and products after 7-day immersion in SIF and Hank’s solution, analyzed by SEM. A thick layer of loose biodegradation products covers the entire active corrosion surface on specimens from both SIF and Hank’s solution, indicating severe corrosion attack. Moreover, biodegradation is much more severe in SIF, showing a rough corrosion surface with irregular ridges (Figure 6a). In contrast, biodegradation products in Hank’s solution are mostly horizontally distributed (Figure 6g). The observed biodegradation severity from surface corrosion morphologies aligns with the biodegradation rate determined by immersion tests: more severe corrosion corresponds to a higher biodegradation rate in SIF compared to that in Hank’s solution. The compositions of biodegradation products were analyzed using EDS. The EDS elemental maps of specimens from SIF are shown in Figure 6c–f. EDS results indicate that the biodegradation products contain Mg, O, Ca, P, and K, with Mg and O predominating and small amounts of P, Ca, and K, on specimens from SIF (Figure 6b) and Hank’s solution (Figure 6h). This suggests the biodegradation products are a mixture of magnesium oxide, magnesium hydroxide, and apatite. The presence of Ca and P on the corroded surface indicates that Ca-P compounds precipitate onto the hydroxide layer as biodegradation progresses.

### 3.3. Antibacterial Activity and Cytocompatibility

**Figure 7:**
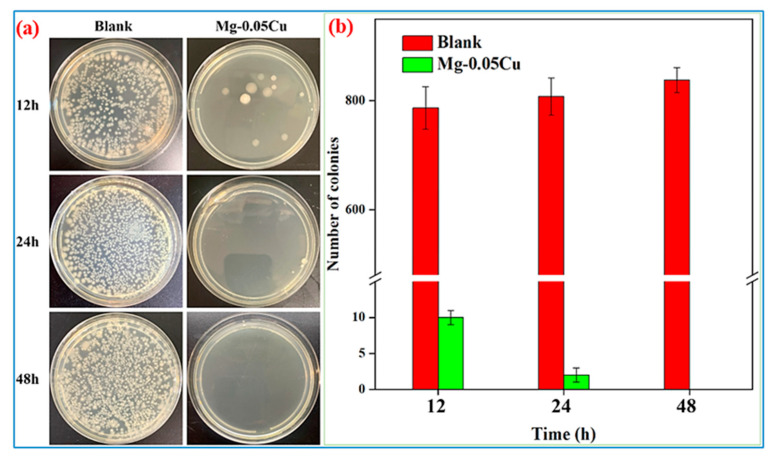
Agar plate images of the SS specimen extracts after 12, 24, and 48 h cultivation with *E. coli* (**a**) and the number of *E. coli* (**b**).

Figure 7a shows agar plate images of extracts from the SS specimens after 12, 24, and 48 h of cultivation with *E. coli*, with a blank group serving as the control. The agar plate for the blank control group is almost completely covered by *E. coli* colonies for every cultivation time. By contrast, the agar plate for the specimen extract group reduced colonies to single-digit numbers in 12 h cultivation, with almost no *E. coli* colonies in 24 h cultivation and 48 h cultivation. The number of *E. coli* CFU/ml in extracts of the blank control group and the specimen group in different time intervals is also shown in Figure 7b. The number of colonies of *E. coli* in the blank control group reaches approximately 789 ± 50 and undergoes little change with increasing time, while the number of colonies of the specimen extract group significantly decreases to 10 ± 1 in 12 h cultivation and further decreases to zero in 24 h cultivation and 48 h cultivation. These data are provided in Supplementary Table S1. The antibacterial rates are calculated by Equation (6):

(6) $$K=\frac{\alpha_{0}-\alpha_{t}}{\alpha_{0}}\times 100\%$$

where *K* is the antibacterial rate, *α*_0_ is the number of bacterial colonies in the blank control group, and *α_t_* is the number of bacterial colonies in the specimen extract group. The obtained antibacterial rates against *E. coli* are 99.3% for 12 h cultivation, and 100% for 24 h cultivation and 48 h cultivation. These results confirm strong antibacterial activity against *E. coli* for the trace-Cu-modified Mg alloy.

**Figure 8:**
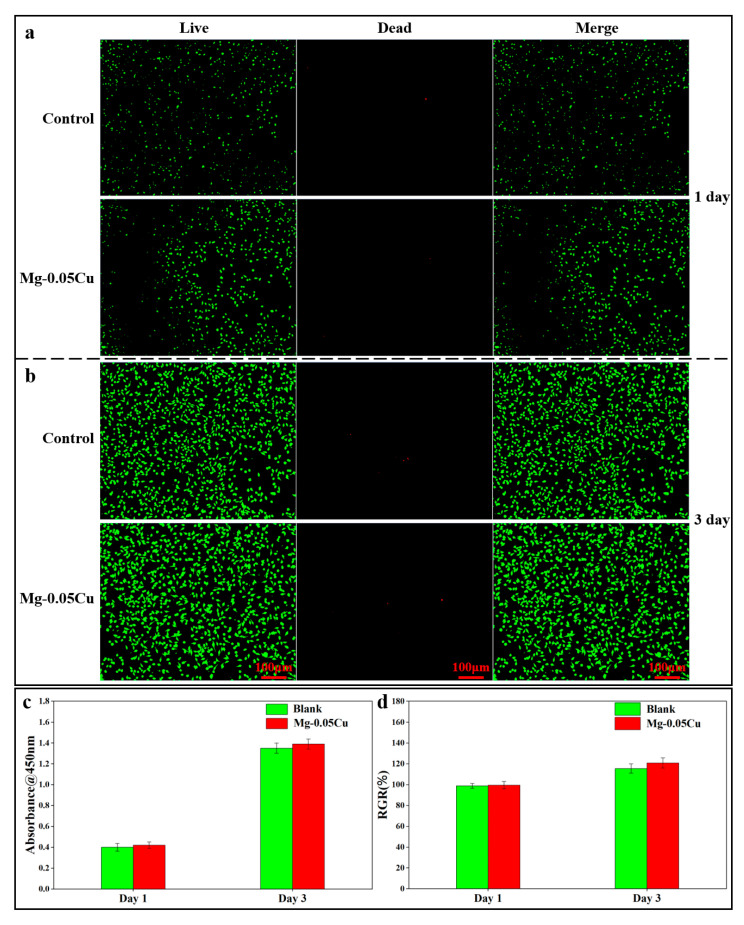
(**a**,**b**) Live/dead fluorescence staining images of L929 cells co-cultured with extracts for 1 day and 3 days. (**c**) CCK-8 assay. (**d**) RGR.

Acceptable cytocompatibility is essential, and in vitro cytotoxicity testing is necessary. A cell viability assay was carried out to evaluate the cytocompatibility. Figure 8a,b show live/dead fluorescence staining images of L929 cells after 1- and 3-day exposure to extracts. After 1 day (Figure 8a), all groups showed predominantly green fluorescent live cells with minimal red fluorescence from dead cells. By day 3 (Figure 8b), both control and extract-treated groups displayed a clear increase in cell density, while the extract-treated group maintained comparable or slightly higher viability with only very few dead cells observed. The relative growth rate (RGR) was calculated by Equation (7):

(7) $$RGR=\left({OD}_{sample}÷{OD}_{blank}\right)\times 100\%$$

where *OD_sample_* is the absorbance of cells treated with sample extract, and *OD_blank_* is the absorbance of cells cultured in the extract-free fresh medium. Figure 8c,d provide CCK-8 assay results and relative growth rate (RGR) data. Quantitative CCK-8 assays and RGR metrics corroborate these observations, collectively confirming the extracts’ excellent cytocompatibility.

## 4. Discussion

### 4.1. Divergent Biodegradation Behavior in SIF vs. Hank’s Solution

The significantly accelerated biodegradation rate of the trace-Cu-modified Mg alloy (Mg-0.05Cu) observed in SIF compared to Hank’s solution is a critical finding, underpinned by fundamental differences in the initial solution chemistry, resultant biodegradation mechanisms, and the nature of the protective/destructive layers formed. This distinct contrast, consistently demonstrated through electrochemical polarization, immersion-derived weight loss, pH evolution, and surface morphology analysis, arises from the interplay of several key factors.

The initial acidic pH of SIF (pH 6.8) creates an inherently more aggressive environment for Mg biodegradation compared to the near-neutral pH of Hank’s solution (pH 7.5). The acidic conditions in SIF significantly accelerate the anodic dissolution reaction (Equation (2)) from the very beginning of exposure. This rapid initial dissolution consumes H^+^ ions, driving the pH upwards sharply. In contrast, Hank’s solution starts closer to neutrality. While biodegradation begins immediately upon immersion (evidenced by the rapid pH surge to ~10.5 within 3 h), the initial driving force for dissolution is thermodynamically less potent than in the acidic SIF environments. The initial reaction involves direct interaction of anions (Cl^−^, CO_3_^2−^, HPO_4_^2−^) with the Mg surface.

The electrochemical polarization data gives direct mechanistic insight. The more negative corrosion potential (*E*_corr_) measured in SIF indicates a higher thermodynamic tendency for the Mg matrix to undergo oxidation (dissolution) compared to Hank’s solution. Crucially, the corrosion current density (*i*_corr_) in SIF (239 μA/cm^2^) was ~4 times higher than in Hank’s solution (61 μA/cm^2^). This translates directly to the significantly higher calculated biodegradation rates (*P*_i_: 5.5 mm/year in SIF vs. 1.4 mm/year in Hank’s solution). This dramatic difference confirms that the kinetics of the anodic dissolution reaction are vastly accelerated in SIF. The acidic environments likely both facilitate the breakdown of any nascent oxide film and enhance the rate of the Mg dissolution reaction itself. The complex compositions of SIF (including organic components like bile salts and enzymes) may also contribute to increased ionic conductivity or alter interfacial processes, further promoting biodegradation compared to the more inorganic Hank’s solution.

**Figure 9:**
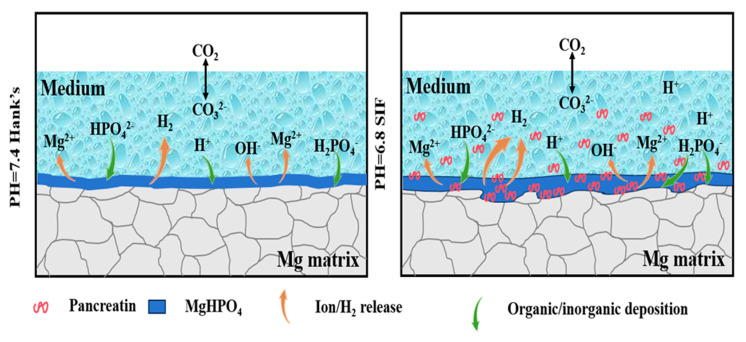
Schematic diagram of corrosion mechanism.

The initial acidity of SIF results in an extremely rapid biodegradation onset. The massive release of Mg^2+^ ions and the concomitant cathodic hydrogen evolution reaction (Equation (3)) generate OH^−^ ions at a very high rate. This overwhelms the buffering capacity of SIF, causing a swift and substantial pH increase, stabilizing at a high value (pH: ~11.8). While Mg(OH)_2_ forms, the rate and intensity of the initial biodegradation appear to disrupt the formation of a compact protective layer. The high stabilized pH itself, reflecting a high concentration of OH^−^ ions, indicates a persistently higher biodegradation rate even after stabilization compared to Hank’s solution. In Hank’s solution, the initial biodegradation surge also causes a rapid pH increase to a peak (pH 11.7 at 24 h). However, the subsequent behavior differs significantly. As immersion continues beyond 24 h, the pH decreases and stabilizes at a lower value (~11.0) than in SIF (~11.8). This decrease is attributed to the formation of denser and more protective surface layers. The presence of Ca and P on the corroded solution promotes the precipitation of Ca-P compounds, which gradually covers the surface, acting as a barrier that isolates the underlying Mg from the solution, slows down the biodegradation reaction, reduces the rate of OH^−^ generation, and consequently lowers the pH. EDS confirmed Ca and P within both the biodegradation products, but the morphology (horizontal distributions in Hank’s solution vs. rough ridges in SIF) suggests that a more cohesive and protective layer formed in Hank’s solution. The degradation mechanism of the trace-Cu-modified Mg alloy is schematically illustrated in Figure 9.

SEM analysis revealed a critical distinction in corrosion morphology and product layer integrity. In SIF, the corrosion surface was extremely rough with irregular ridges, covered by a thick layer of loose biodegradation products. This non-adherent loose structure provides minimal protection, allowing continued aggressive attack by the fluid. The rapid biodegradation rate prevents the formation of a coherent barrier. In Hank’s solution, while biodegradation occurred, the damage appeared more “horizontally distributed”, suggesting a more uniform front beneath the surface layer, although degrading. The observed pH decrease over time strongly correlates with the formation of denser biodegradation products (Mg(OH)_2_ + Ca-P compounds) that act as a more effective diffusion barrier, slowing ion transport and reducing the biodegradation rate over extended periods. This difference in product layer protectiveness directly contributes to the consistently lower *P*_w_ values observed in Hank’s solution throughout the immersion test. The immersion weight loss data consistently showed higher rates (*P*_w_) in SIF than in Hank’s solution at every test timepoint. Both solutions exhibited a decrease in *P*_w_ over time, reflecting the influence of surface layers. However, the magnitude of the rate and the severity of surface damage (SEM) were always significantly greater in SIF. This confirms that while some degree of protection develops in both environments, the protective layer in SIF is fundamentally less effective due to the combination of initial aggressiveness and the nature of the products formed.

The pronounced difference in biodegradation rate between SIF and Hank’s solution for the trace-Cu-modified Mg alloy stems primarily from the synergistic effect of the initial acidic pH and complex compositions (including organic components like bile salts and enzymes) of SIF and the resultant inability to form a cohesive, protective biodegradation product layer. The low initial pH and the complex compositions of SIF provide a strong thermodynamic and kinetic driving force for rapid dissolution. This intense initial attack hinders the development of a stable passivating film and leads to the formation of a thick, loose, and non-protective layer of biodegradation products (primarily Mg(OH)_2_ with some Ca-P). Consequently, the biodegradation process remains highly active, sustaining a high pH and biodegradation rate. In contrast, the near-neutral start in Hank’s solution allows for a more controlled initial reaction, facilitating the earlier formation of a denser, more protective composite layer (Mg(OH)_2_ + Ca-P compounds). This layer, while not halting biodegradation, significantly retards it over time, resulting in lower overall biodegradation rates and less severe surface damage. These findings highlight the critical importance of using physiologically relevant media like SIF for evaluating Mg-based biomaterials intended for gastrointestinal applications, as standard physiological solutions like Hank’s significantly underestimate the material’s biodegradation rate, specifically in more aggressive environments.

### 4.2. Antibacterial Mechanism of Trace-Cu-Modified Mg Alloy

The potent antibacterial efficacy demonstrated by the trace-Cu-modified Mg alloy (Mg-0.05Cu) against *E. coli*, achieving near-total eradication (99.3% at 12 h and 100% at 24 h and 48 h), is a significant finding. While Mg itself possesses inherent antibacterial properties related to its biodegradation (alkalization), the remarkably enhanced and rapid bactericidal effect strongly implicates Cu modification as playing a critical role. The synergy between Mg biodegradation and controlled Cu^2+^ ion release creates a uniquely hostile environment for bacteria, surpassing the inherent activity of pure Mg. Here, the likely mechanisms underpinning this potent antibacterial action are explored, integrating observations from microstructure, biodegradation behavior, and biological testing.

The antibacterial mechanism depends on the controlled release of Cu^2+^ ions into the microenvironment. Microstructural characterization (SEM-EDS, Figure 1) unequivocally identified the secondary-phase particles in both AC and SS conditions as Cu-rich intermetallic phases, primarily composed of Mg_2_Cu. The accelerated biodegradation of the Mg matrix in SIF, as extensively documented in the results (higher *i*_corr_, *P*_i_, and *P*_w_, rapid and significant pH surge, severely biodegraded surface morphology), provides the driving force for Cu^2+^ ion release. As the α-Mg matrix dissolves rapidly in the acidic-to-alkaline shifting environment of SIF, the Mg_2_Cu intermetallic particles are preferentially attacked and dissolved. This galvanic coupling effect ensures a sustained release of Cu^2+^ ions concurrent with Mg biodegradation. EDS elemental maps (Figure 1(a_3_–a_5_,b_3_–b_5_)) visually confirmed Cu’s localization within the secondary phases. Their dissolution during biodegradation releases Cu^2+^ ions into the surrounding fluid, directly evidenced by the presence of Cu^2+^ ions in the extracts used for antibacterial testing. The biodegradation rate in SIF is much higher than in Hank’s solution, implying a potentially higher flux of Cu^2+^ ions released in SIF within the same timeframe, contributing to the bactericidal effect.

**Figure 10:**
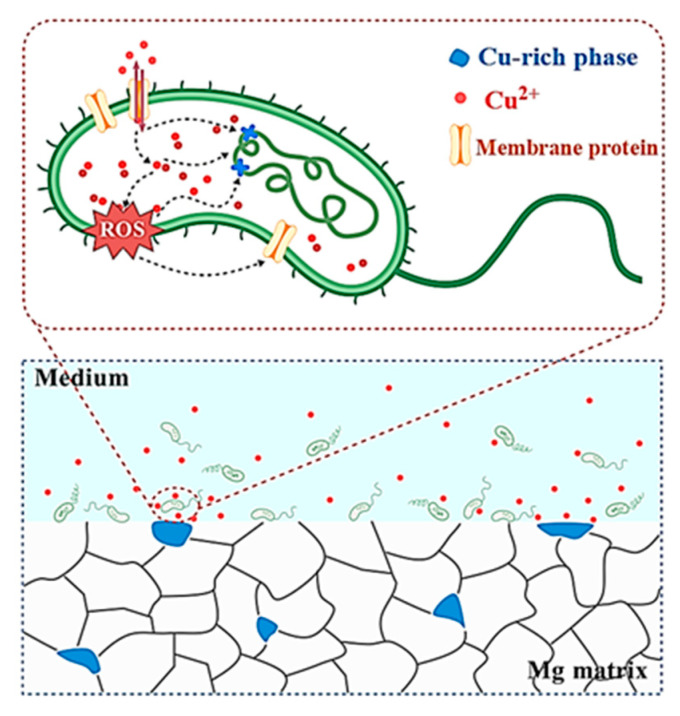
Schematic diagram of antibacterial mechanism.

The ions produced during the biodegradation process are released into physiological environments. The Cu-rich phases in the trace-Cu-modified Mg alloy are responsible for the release of Cu^2+^ ions during biodegradation, driving its sustained antibacterial effect, as demonstrated above. Specifically, a bactericidal rate of 100% against *E. coli* was achieved after 24 h and 48 h of cultivation. The antibacterial property of Cu is well established and can be attributed to the following mechanisms: Released Cu^2+^ ions exert antibacterial effects by disrupting bacterial cell membranes and inducing oxidative stress [31,32,33,34]. Specifically, (i) Cu^2+^ ions bind to negatively charged components (e.g., lipopolysaccharides, phospholipids) on bacterial cell walls and membranes. This disrupts membrane integrity, increases permeability, causes leakage of vital cellular contents, and ultimately leads to cell lysis. The rapid reduction in CFU counts observed within 12 h confirms membrane damage as an early mechanism. (ii) Cu^2+^ ions participate in Fenton-like reactions within bacterial cells, catalyzing the conversion of endogenous hydrogen peroxide (H_2_O_2_) or superoxide (O_2_^−^) into highly toxic hydroxyl radicals (•OH) and other reactive oxygen species (ROS). This oxidative burst overwhelms bacterial antioxidant defenses, causing severe oxidative damage to lipids, proteins, and DNA. The antibacterial mechanism from Cu^2+^ ion release in the trace-Cu-modified Mg alloy is schematically illustrated in Figure 10.

The antibacterial action of Cu^2+^ ions does not operate in isolation, being strongly augmented by the local environment created by Mg biodegradation. The rapid biodegradation of Mg generates a large amount of OH^−^ ions, causing a sharp and significant rise in the local pH (∼11.8 in SIF, ∼11 in Hank’s solution). This highly alkaline environment is inherently detrimental to many bacteria, including *E. coli*, damaging their cell membranes and disrupting intracellular pH homeostasis [35,36,37,38,39,40]. The alkaline conditions may also facilitate the precipitation of copper compounds directly onto bacterial cells. The kinetics of the antibacterial effect are remarkable. The dramatic reduction to single-digit CFU counts within 12 h and complete eradication by 24 h indicate a rapid and potent mode of action. Even with only trace Cu (∼0.05 wt.%), the biodegradation-driven dissolution releases Cu^2+^ ions at a concentration locally exceeding the minimum inhibitory/bactericidal concentration for *E. coli* within a short timeframe. The continuous biodegradation ensures sustained release. The combination of Cu^2+^ ions and the alkaline/high-osmolarity environment acts synergistically. Cu^2+^ ions might inflict initial membrane damage, making cells more susceptible to the denaturing effects of high pH and ROS. Conversely, the alkaline environment might enhance the penetration or reactivity of Cu^2+^ ions. This synergy likely achieves bactericidal effects faster and at lower effective Cu concentrations than either factor alone. While biodegradation products form, the observed loose morphology suggests that they do not completely seal the surface or prevent ongoing Cu^2+^ ion release from underlying or freshly exposed Mg_2_Cu particles, maintaining the antibacterial activity over time (evidenced by sustained 100% efficacy at 24 h and 48 h).

Crucially, this potent antibacterial effect is achieved without compromising cytocompatibility, as demonstrated by the excellent viability and proliferation of L929 cells exposed to the extracts (Figure 8). This indicates a selective toxicity favoring prokaryotic cells (bacteria) over eukaryotic mammalian cells under the tested conditions. Possible mechanisms include the following: (i) Bacterial cells, lacking the complex protective structures of mammalian cells, are generally more vulnerable to membrane disruption and ROS. (ii) The local concentration of Cu^2+^ and OH^−^ near the degrading material surface is likely highest, primarily affecting adherent bacteria. Mammalian cells exposed to the diluted extract in the bulk medium experience lower, nontoxic concentrations. (iii) Mammalian cells possess more sophisticated antioxidant systems and repair mechanisms to counter oxidative stress compared to bacteria.

The potent and rapid antibacterial activity of the trace-Cu-modified Mg alloy against *E. coli* derives primarily from the synergistic action of Cu^2+^ ions released from the dissolving Mg_2_Cu intermetallic phase and the hostile local microenvironment generated by rapid Mg biodegradation (high pH). The accelerated biodegradation in SIF ensures a sufficient and sustained flux of Cu^2+^ ions. These ions inflict multi-target damage on bacteria, including membrane disruption and ROS generation. The highly alkaline conditions resulting from Mg biodegradation further potentiate this effect by damaging bacterial membranes and potentially enhancing Cu activity. This synergy leads to rapid bacterial death, achieving sterilization within 24 h. Importantly, this potent antibacterial efficacy is achieved while maintaining excellent cytocompatibility, highlighting trace Cu alloying as a highly effective strategy for developing biodegradable Mg implants with intrinsic infection-fighting capabilities. This is particularly valuable for applications exposed to challenging environments like the gastrointestinal tract. The synergistic antibacterial performance and confirmed biocompatibility constitute promising functional advantages.

## 5. Conclusions

The as-cast Mg-0.05Cu alloys exhibit coarse primary α-Mg grains with Cu-rich secondary phases identified as Mg_2_Cu intermetallics via SEM-EDS. Solid solution treatment reduces but does not completely eliminate Mg_2_Cu particles, confirming their role as Cu reservoirs.

The biodegradation in SIF is faster than in Hank’s solution, as evidenced by electrochemical and immersion testing. The biodegradation rates determined by electrochemical and immersion testing for the trace-Cu-modified Mg alloy in SIF are consistent with reported values for Mg alloys in similar media, as are those in Hank’s solution.

The initial acidity of SIF drives rapid anodic dissolution, disrupting protective layer formation. Biodegradation products with loose non-protective irregular ridges (Ca-P/Mg(OH)_2_) form in SIF due to aggressive initial biodegradation, enabling sustained biodegradation. In contrast, horizontally distributed Ca-P/Mg(OH)_2_ corrosion layers in Hank’s solution retard biodegradation over time.

Mg-0.05Cu achieves a 99.3% antibacterial rate against *E. coli* within 12 h and 100% eradication at 24–48 h, while demonstrating excellent biocompatibility with L929 cells (>95% viability and proliferation maintained over 3 days). Synergy between sustained Cu^2+^ release (from dissolving Mg_2_Cu) and a high-pH microenvironment (from Mg biodegradation) is responsible for rapid bactericidal action. This validates selective toxicity favoring bacterial eradication over mammalian cell harm.

The harsh environment of SIF critically accelerates biodegradation versus conventional Hank’s solution, underscoring the necessity of physiologically relevant media for gastrointestinal implant evaluation. The synergistic antibacterial performance and confirmed biocompatibility achieved through trace Cu alloying (0.05 wt.%) constitute key functional advantages, offering a promising strategy for developing infection-resistant biodegradable Mg implants in physiologically relevant intestinal environments.

## Figures and Tables

**Table 1:** Compositions of the as-cast Mg-Cu alloy (wt.%).

Nominal Composition	Actual Composition				
	Cu	Fe	Si	Ni	Mg
Mg-0.05Cu	0.057	<0.005	0.012	<0.001	Balance

## Data Availability

The original contributions presented in the study are included in the article; further inquiries can be directed to the corresponding author.

## Supplementary Materials

The following supporting information can be downloaded at: https://www.mdpi.com/article/10.3390/jfb16090344/s1, Table S1: Raw data of antibacterial evaluation against *E. coli*.
