# Supplementary material for: Antibacterial Activity of a Trace-Cu-Modified Mg Alloy in Simulated Intestinal Fluid
Source: J Funct Biomater. 2025 Sep 12;16(9):344. doi: 10.3390/jfb16090344 (PMC12470997; doi:10.3390/jfb16090344)
Supplement: Supplementary file 1 [file jfb-16-00344-s001.zip › jfb-3839381-supplementary.pdf]

Table S1. Raw data of antibacterial evaluation against *E. coli*.

		Blank			Mg-0.05Cu		
Dilution plated		10^{-5}			10^{-5}		
Count	12h	730	800	837	10	9	11
	24h	760	815	859	4	6	7
	48h	795	877	843	0	0	0
Real CFU/mL	12h	7.3×10^8	8.0×10^8	8.4×10^8	1.0×10^7	9.0×10^6	1.1×10^7
	24h	7.6×10^8	8.2×10^8	8.6×10^8	4.0×10^6	6.0×10^6	7.0×10^6
	48h	8.0×10^8	8.8×10^8	8.4×10^8	＜LOD	＜LOD	＜LOD

Note: "LOD (limit of detection) = 1 / (volume plated × dilution factor) = 1×10^6 CFU/mL under these conditions."
